# The Correlation between miR-122 and Lipoprotein Lipase Expression in Chronic Hepatitis C Patients

**DOI:** 10.1155/2018/6348948

**Published:** 2018-07-24

**Authors:** Malgorzata Sidorkiewicz, Martyna Grek-Kowalinska, Anna Piekarska

**Affiliations:** Medical University of Lodz, Poland

## Abstract

Chronic HCV infection is strictly associated with host lipid/lipoprotein metabolism disorders. The study aimed to analyze the relationship between viral load, lipid profile, IFN*γ*, and the expression of miR-122 and LPL in the liver and PBMCs. Sera, PBMCs, and matching liver biopsies from 17 chronic hepatitis C patients were enrolled in this study. Collected data shows that liver (not PBMCs) miR-122 expression is positively correlated with HCV RNA load and IFN*γ* and reversely with LPL expression in CHC patients. Presented, for the first time, in this study, the reverse correlation of miR-122 and LPL expression in liver; miR-122 and LPL seem to be important factors of CHC infection.

## 1. Introduction

Hepatitis C virus (HCV), with an estimated over 185 million people infected worldwide [1], is the major etiological cause of chronic hepatitis. Chronic hepatitis C (CHC) is directly associated with development of liver cirrhosis and hepatoma. HCV RNA triggers production of interferon gamma (IFN*γ*) but is often abrogated in CHC patients [2]. Reduced IFN*γ* level implicates an immunological impairment that in turn consolidates the state of chronicity.

HCV infection affects host lipids/lipoproteins homeostasis. Alteration of metabolism seems to be essential for the HCV entire “life cycle”: entry, replication, and the secretory pathway [3]. Circulating HCV virions are associated with VLDL and LDL, designated as lipo-viro-particles. Lipoprotein lipase (LPL) that catalyzes triglyceride (TG) hydrolysis of chylomicrons and VLDL has been identified as a promising host factor against HCV infection [4]. Lipo-viro HCV particles constitute effective substrate for LPL; thus reduction of HCV infectivity can be achieved by modifying the virion-associated lipid composition [5]. Additionally, LPL increases the binding capacity of HCV particles and reduces infectivity of the virus, possibly by blocking virions on the surface of cells [6]. Also, LPL-induced peroxisomal proliferators-activated receptor-*α* (PPAR-*α*) signaling pathway activation was suggested as a factor that suppresses HCV infection [7].

Previous reports underscore the importance of miR-122 in liver biology and in the control of HCV infection (for review [8]). miR-122 exerts in vitro a positive effect on HCV replication through a direct interaction with 5'UTR region of viral genome. Inhibition of miR-122 reduces serum cholesterol level [9]. HCV RNA sequences were detected in vivo, not only in hepatocytes but also in fresh peripheral blood mononuclear cell (PBMC) preparations from HCV-infected patients as well as cultured PBMCs [10]. In previous studies we showed not only changes in serum lipid profile in CHC patients but also in cholesterol expression in PBMCs [11]. Neither this lipid's alteration nor HCV RNA presence in cells was related to miR-122 expression in PBMCs.

This study compares miR-122 and lipoprotein lipase expression in the liver and PBMCs and investigates how these parameters are associated with lipid profile, with HCV load and IFN-gamma, revealing the reverse correlation between lipoprotein lipase and miR-122 expression in the liver of CHC patients. To the best of our knowledge this study represents the first attempt to evaluate the relationship between miR-122 and LPL expression.

## 2. Materials and Methods

### 2.1. Participant Enrollment

This study recruited 17 patients with CHC (genotype 1) and 26 healthy donors (HD). Blood samples obtained from both groups, CHC patients and healthy donors, were used for PBMCs and sera isolation. Serum HCV RNA was detected by the Amplicor HCV test, version 2.0 (Roche Diagnostics). In case of CHC patients, matching human liver specimens from fine needle biopsies were collected. The study was approved by the Bioethical Committee of the Medical University of Lodz (RNN/93/07/kB); signed informed consent from all participants was obtained.

### 2.2. Determination of Biochemical Parameters in Sera and PBMCs

We followed the methods of Sidorkiewicz et al. 2017 [12]. Serum total cholesterol (TC), high-density lipoprotein-cholesterol (HDL-C), low-density lipoprotein-cholesterol (LDL-C), and triglycerides (TG) were measured enzymatically in Olympus AU 640 and the kits of Olympus. The IFN*γ* concentration in sera was measured using Human IFN*γ* ELISA kit (BioVendor).

Intracellular cholesterol level in PBMC (IC) was evaluated using the cholesterol assay kit, Cholesterol CHOD-PAP (BIOLABO S.A., France) as per manufacturer's recommendation, and the results were normalized to the protein concentration in cell lysates and presented as relative expression of (r.e. IC).

### 2.3. miR-122 Analysis

For miR-122 analysis, RNA fraction <200 nt was extracted from PBMCs, using mirVana miRNA Isolation Kit (Ambion), and total RNA from biopsy samples using TRIzol Reagent (Invitrogen) according to the manufacturer's instructions. The reverse transcription (RT) was done, on 10 ng of RNA using the TaqMan MicroRNA Assay specific for miRNA-122 (Applied Biosystems). RT product (3.3 *μ*L) was used in Real-Time PCR with miRNA-122-specific primers/probe mix and TaqMan Universal PCR Master Mix (Applied Biosystems). The reaction was performed on the ABI Prism 7900 Fast Real-Time PCR System (Applied Biosystems). Relative expression (r.e.) of miR-122 presents the mean Ct value for miRNA-122 Ct, calculated from triplicate reactions and normalized to average Ct values, obtained for snRNA U6.

### 2.4. Determination of LPL Expression

LPL expression was determined by Western Blotting. A total of 50 *μ*g of protein from PBMCs lysates was subjected to SDS-PAGE, transferred into nitrocellulose, and incubated with rabbit anti-LPL primary antibodies (sc-32885, Santa Cruz Biotechnology, dilution 1:300) and secondary goat anti-rabbit antibodies (sc-2004, Santa Cruz Biotechnology, dilution 1:4000). As a control, goat antibodies against *β*-actin (sc-1615, Santa Cruz Biotechnology, dilution 1:300) were used with secondary rabbit anti-goat (1:10000) from Sigma. The positive bands were detected using enhanced chemiluminescence system (ECL) and were quantitatively analyzed using the Bio-Rad Quantity One System. Relative expression (r.e.) of LPL was calculated by normalization to the actin expression for each sample.

### 2.5. Statistical Analysis

Statistical analysis was performed with STATISTICA 8,0 PL software (StatSoft). Groups were compared using the Mann–Whitney* U* test. Spearman's correlation analysis was applied to measure the strength of relationship between parameters. Differences were considered statistically significant at p<0,05.

All origin data supporting the results reported in this paper are collected and available in the Department of Medical Biochemistry.

## 3. Results

This study recruited 17 patients with CHC (genotype 1) and 26 healthy donors (HD). General characteristics of studied subjects are included in Table 1. As presented, the level of total cholesterol, LDL, and HDL fractions in sera as well as intracellular cholesterol level in PBMCs were significantly lower in CHC patients compared to healthy donors. Similarly, serum IFN*γ* was significantly lower in CHC patients than in HD. Observed decreased level of triglycerides in CHC patients was not statistically significant. Comparison of LPL and miR-122 expression in PBMCs showed, for both parameters, a higher expression in cells from CHC patients than from HD.

Spearman rank correlation analysis performed on data collected from CHC patients (Table 2) indicated strong reverse correlation for miR-122 expression between liver and PBMCs as well as for LPL expression between these two origins. miR-122 expression in liver was positively correlated with serum HCV RNA load and IFN*γ* and reversely correlated with hepatic LPL expression. We found also the tendency for hepatic LPL expression to be reversely correlated with viral load. Neither for miR-122 nor for LPL expression in PBMCs of CHC patients, significant correlation with HCV RNA load was revealed. Additionally, in the group of healthy donors, strong reverse correlation was found between serum IFN*γ* and both TC (r= -0,52, p=0,008) and LDL (r= -0,42, p=0,039), not detected in the group of CHC patients.

## 4. Discussion

The strategic role of hepatitis C virus in manipulating of host lipids/lipoproteins metabolism was described [3] as essential for the entry, secretion, and replication of virus. Our results showed that TC, LDL, and HDL fractions, as well as intracellular cholesterol level in PBMCs, were significantly decreased in CHC patients compared to healthy donors. These data remain in line with previous observations that associates CHC infections not only with liver steatosis but also with decreased cholesterol expression [13, 14]. HCV may modulate lipid metabolism in host cells in order to create a more hospitable environment for its own “life cycle”.

Concerning IFN, we observed that serum IFN*γ* level was significantly reduced in CHC patients compared to healthy donors, which remains in agreement with earlier reports [15, 16]. Additionally, we found that serum IFN*γ* was reversely correlated with total cholesterol and LDL level, but only in the group of healthy donors. Lack of such a relationship in the group of CHC patients may suggest perturbation between cholesterol metabolism and IFN*γ* signaling. It was shown by others [17] that the alteration of cholesterol pathway may abrogate the IFN-gamma-dependent immune response.

Lipoprotein lipase, which plays a main role in lipoprotein/lipid homeostasis, emerged recently as a host-protecting factor against HCV infection. Indeed, a strong tendency to opposite correlation between HCV RNA load and hepatic expression of lipoprotein lipase seems to confirm these observations in our study. Lack of such a relationship for LPL in PBMCs can be explained by opposite correlation between LPL expression in liver and PBMCs. Interestingly, while LPL may regulate TG metabolism, no correlation was found between LPL expression and TG level.

According to the suggested role of miR-122 in HCV replication [8], our results demonstrated the positive correlation between the HCV RNA load and miR-122 expression in liver (not in PBMCs). Like in case of LPL, miR-122 showed also opposite expression in the liver and in PBMCs. Hepatic miR-122 was reversely correlated with the expression of lipoprotein lipase. It is especially interesting observation as liver-specific miR-122 is known to promote hepatitis C virus replication and lipoprotein lipase to protect the host from HCV infection.

As was mentioned earlier, LPL may suppress HCV infectivity by the breakdown of viral lipid coat and bridging between HCV and hepatocytes that blocks the viral entry to the cell [4, 6]. The third mechanism of anti-HCV action of LPL is based on activation of peroxisomal proliferators-activated receptor-*α* (PPAR-*α*) signaling pathway [7]. After LPL hydrolysis free fatty acids create ligands for this pathway in target cells. That in turn may cause an antiviral action in infected patients via reduction of viral assembly and secretion. Independently of LPL activity, the same pathway may be triggered directly by alteration of miR-122 expression. The study of Gatfield [18] demonstrated that the knockdown of miR-122 expression resulted in the activation of PPAR-*α* signaling pathway.

Additionally, we found that miR-122 expression in liver (not in PBMCs) is positively correlated with IFN*γ* concentration. Interestingly, it was found earlier that IFN*γ* is involved in lipoprotein lipase inhibition, at the level of LPL gene transcription [19]. Thus, we cannot exclude that, in CHC patients, IFN-gamma may participate in reducing of LPL expression along with miR-122.

In conclusion, this study provides evidence for opposite expression of both miR-122 and lipoprotein lipase in liver and PBMCs. Hepatic miR-122 expression in CHC patients seems to be of high importance for HCV infection which was evidenced by strong positive relationship between miR-122 and HCV RNA load. The reverse relationship between miR-122 and lipoprotein lipase in liver suggests that the modulation of lipoprotein lipase may be another manifestation of the proinfective action of miR-122. Therefore, a future study attempting to explain the mechanism of cross-reactivity between miR-122 and LPL is worth considering.

## Figures and Tables

**Table 1:** Comparison of analyzed parameters in the group of CHC patients and healthy donors.

Analyzed parameters (mean values)	CHC patients	Healthy donors	p value
Number (M/F)	17 (13/4)	26 (15/11)	n.a.
Age (ys)	22,7	23,2	n.a.
ALT (IU/ml)	69,18	20,6	0,003
IFN gamma (pg/ml)	4,41	5,52	0,015
TC (mmol/L)	2,55	3,61	<0,0001
LDL-C (mmol/L)	1,4	2,03	0,003
HDL-C (mmol/L)	0,8	1,09	0,002
TG (mmol/L)	0,77	1,1	0,16 (ns)
r.e. IC (PBMCs)	1,12	1,76	<0,0001
HCV RNA load (IU/ml)	3,79 x 10^5^	n.a.	n.a.
r.e. LPL (PBMCs)	2,37	1,32	0,008
r.e. LPL (Liver)	3,12	n.a.	n.a.
r.e. miR-122 (PBMCs)	0,88	0,76	0,001
r.e. miR-122 (Liver)	1,15	n.a.	n.a.

ALT: alanine aminotransferase; F: female; M: male; n.a.: not analyzed; ns: not statistically confirmed result; r.e.: relative expression; ys: years.

**Table 2:** The list of correlations (Spearman rank correlation test) found for data obtained by the analysis of sera (S), liver biopsies (L), and PBMCs (P) samples collected from CHC patients.

Compared parameters	Correlation coefficient value	P value
miR-122 (L) vs HCV RNA (S)	+0,63	0,006
miR-122 (L) vs IFN *γ* (S)	+0,54	0,024
miR-122 (P) vs HCV RNA (S)	-0,81	0,026
miR-122 (P) vs miR-122 (B)	-0,93	0,001
LPL (L) vs LPL (P)	-0,56	0,028
LPL (L) vs miR-122 (B)	-0,47	0,014
LPL (L) vs HCV RNA (S)	-0,31	0,06*∗*
LPL (P) vs HCV RNA (S)	+0,42	0,036

*∗* - indicates a tendency.

## Data Availability

The data used to support the findings of this study are available from the corresponding author upon request.
